# Fibrillin-1 Gene Variant p.Gly1754Ser Associated With Weill-Marchesani Syndrome Type 2: A Case Report

**DOI:** 10.7759/cureus.69448

**Published:** 2024-09-15

**Authors:** Parag M Tamhankar, Pramila Menon, Shailaja V Mane, Aarthi Muthu Kumar

**Affiliations:** 1 Pediatrics, Dr. D.Y. Patil Medical College, Hospital and Research Centre, Dr. D.Y. Patil Vidyapeeth (Deemed to be University), Pune, IND

**Keywords:** acromelic dysplasia, autosomal dominant inheritance, ectopia lentis, exome sequencing, fbn1 gene, insilico analysis, joint stiffness, rare skeletal dysplasia, short stature (ss), weil marchesani syndrome

## Abstract

Weill-Marchesani syndrome (WMS) is a rare connective tissue disorder characterized by severe short stature, small hands and feet, joint stiffness, eye abnormalities such as microspherophakia, ectopia of lenses, severe myopia, glaucoma, and heart defects. This case study describes a nine-year-old female child with WMS syndrome type 2 and heterozygous pathogenic variant p.Gly1754Ser in the fibrillin-1 gene, identified on whole exome sequencing. Two individuals with WMS with the p.Gly1754Ser variant have been previously reported in the medical literature. The present case is the fourteenth case of WMS type 2 with fibrillin-1 gene mutation in the medical literature, to the best of the author’s knowledge.

## Introduction

Mutations in the fibrillin-1 (FBN1) gene are associated with a range of phenotypes, such as Marfan syndrome (MFS); Marfan lipodystrophy syndrome; mitral valve prolapse, aortic dilatation, skeletal and skin findings, short-sightedness syndrome; stiff skin syndrome; acromicric dysplasia (AMD); geleophysic dysplasia type 2 (GLD); Weill-Marchesani syndrome (WMS) type 2; and familial ectopia lentis syndrome. AMD, GLD, and WMS, collectively called acromelic dysplasias, have overlapping phenotypes presenting as shortness of height and stiff joints. However, the facial phenotype is considered distinct. Lens dislocation and small spherical lenses occur in WMS and are absent in AMD and GLD [1,2]. This case study describes a nine-year-old female child with a WMS phenotype and heterozygous mutation p.Gly1754Ser in the FBN1 gene. This mutation has been reported twice in families with individuals with the WMS phenotype [3]. The present case is the third reported case of WMS with the p.Gly1754Ser mutation and the fourteenth WMS case with the FBN1 gene mutation in the medical literature, to the best of the author’s knowledge. WMS is a rare genetic disorder with the following features: short height, small hands and feet, stiff joints, small lenses, lens dislocation, shortsightedness, increased intra-ocular pressure, and heart defects. Dr. Georges Weill from France was the first to note the co-occurrence of small hands, small lenses, and lens dislocation in 1932 [4]. In 1939, Dr. Oswald Marchesani from Germany explained the common pathogenesis of these symptoms and denoted them as a distinct syndrome [5]. There are four types of WMS. WMS syndrome types I, III, and IV are inherited in an autosomal recessive manner and caused by mutations in the ADAMTS10, LTBP2, and ADAMTS17 genes, respectively; type II is dominant and caused by a mutation in the FBN1 gene [6]. 

## Case presentation

**Figure 1:**
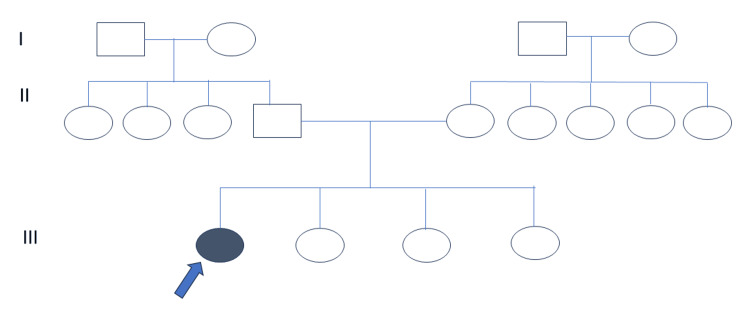
Pedigree of the family showing the index case and unaffected siblings and parents

A nine-year-old female child, born of a non-consanguineous marriage and the eldest of four siblings, presented with shortness of height noticed when she was three years old (Figure 1).

The patient had complained of stiff joints for the past year. She had a history of lens extraction done for bilateral lens dislocation at six years old. She was born at term in a vaginal delivery, and her birth weight was 2.5 kg. The child achieved normal mental and motor milestones and was enrolled in school with average scholarly performance.

**Figure 2:**
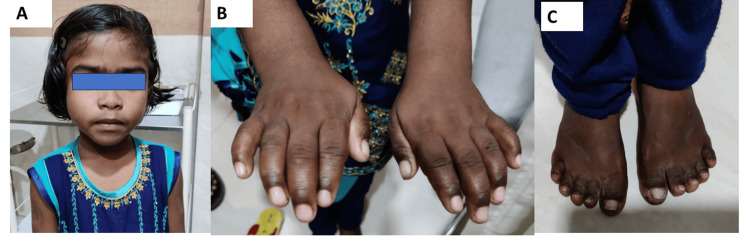
Clinical photos of the face, hand, and feet of the affected index case Dysmorphic facial features (Figure 2A), small hands (Figure 2B), and feet (Figure 2C) are seen. Written informed consent has been obtained for the publication of facial photographs.

She was proportionately short with a height of 95 cm (z score was minus 6.3) and an upper segment (47 cm) to lower segment (48 cm) ratio of 0.97. The father’s height was 168 cm, the mother’s height was 155 cm, and the mid-parental height was 161.5 cm. Her weight was 15 kg (z score was minus 3.5). She had round facies, bushy eyebrows, midfacial hypoplasia, a long philtrum, and a broad lower lip (Figure 2). She had acromelia (Figure 2). Additionally, she had stiffness in the joints of her hands and elbows, and she was unable to make a fist or flex her elbows completely (Figure 2).

**Figure 3:**
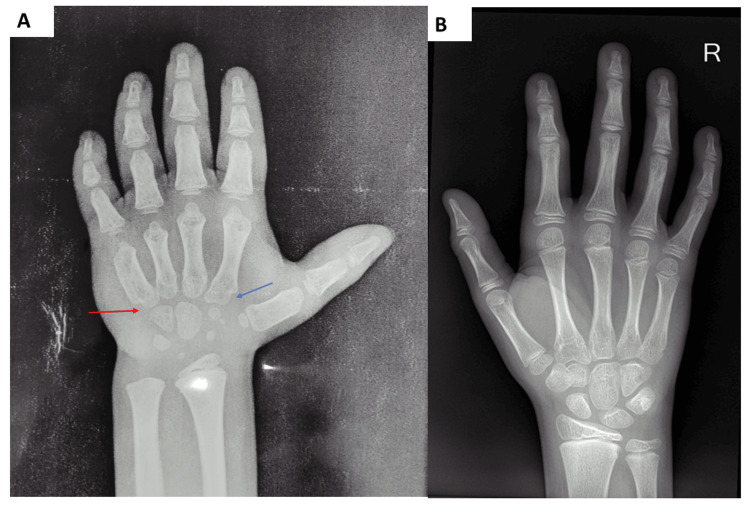
X-ray of a hand with wrist anteroposterior view of the index case (Figure 3A) and the hand of a healthy child of the same age (Figure 3B) The X-ray of the index case (Figure 3A) showed short bones in the hand; conical epiphyses of the phalanges; small round upper epiphyses of the metacarpals; internal notching of the second metacarpal (Figure 3A, blue arrow); and external notching of the fifth metacarpal (Figure 3A, red arrow).

An X-ray of the child’s hand with an anteroposterior view of the wrist, in comparison to that of a healthy child of the same age, showed short bones of the hand; conical epiphyses of the phalanges; small, round upper epiphyses of the metacarpals; internal notching of the second metacarpal; and external notching of the fifth metacarpal. The bone age was six years (Figure 3).

Her vision was low, with the best corrected visual acuity being 6/24 in the right eye and 6/12 in the left eye. The intraocular pressures were normal. The sexual maturity rating was prepubertal (Tanner stage I). A cardiac examination confirmed a murmur and a two-dimensional echocardiography showed an atrial septal defect. The electrocardiogram was normal. Examination of parents and other siblings revealed normal findings. Genetic testing was advised to rule out the clinical diagnosis of WMS. A karyotype test was done to rule out Turner syndrome, and the findings were normal, 46, XX. Whole exome sequencing was done using the Illumina sequencing system (Illumina Inc., CA, USA) at a mean coverage greater than 80-100X and a read quality greater than Q20. A heterozygous variant was identified in the FBN1 gene with genomic coordinates chr15:48460282C>T (GRCh38 format) or c.5260G>A, leading to the missense mutation p.Gly1754Ser. The variant was likely pathogenic as per the American College of Medical Genetics (ACMG) pathogenicity criteria by Franklin online software (https://franklin.genoox.com/clinical-db/variant/snp/chr15-48460282-C-T-hg38).

The following ACMG pathogenicity criteria were fulfilled: pathogenic moderate PM1: nontruncating nonsynonymous variant is located in a mutational hot spot or critical and well-established functional domain (located in an exon hotspot, seven pathogenic, or likely pathogenic reported variants were found in a 48 base-pair region surrounding this variant in exon 43 within the region 48460269-48460317 without any missense benign variants); pathogenic moderate PM2: absent from the genome aggregation database; pathogenic moderate PM5: different amino acid as a known pathogenic variant (chr15:48460281:C>A or c.5261G>T or p.Gly1754Val (Clinvar database ID 2946404) is reported as likely pathogenic in Clinvar and chr15:48460281:C>T or c.5261G>A or p.Gly1754Asp (Clinvar database ID 2570580) is reported as likely pathogenic in Clinvar database); pathogenic supporting PP2: missense variant in a gene with low rate of benign missense mutations and for which missense mutation is a common mechanism of a disease; pathogenic supporting PP3: for a missense or a splicing region variant, computational prediction tools unanimously support a deleterious effect on the gene; pathogenic supporting PP5: reputable source recently reports a variant as pathogenic, but the evidence is not available to the laboratory to perform an independent evaluation.

The predictions of the pathogenicity of the variant by various in-silico software are given in Table 1.

**Table 1:** Results of analysis of the p.Gly1754Ser variant by various in-silico software The in-silico tools showed that when the variant p.Gly1754Ser is deleterious, the higher the scores, the more significant the prediction. The Splice AI tool showed that the variant did not change the splicing mechanism.

Name of in-silico software	Prediction of variant	Score
Franklin	deleterious	0.73
Rare Exome Variant Ensemble Learner (REVEL)	deleterious	0.74
AlphaMissense	deleterious	0.898
Evolutionary Model of Variant Effect (EVE)	deleterious	0.64
Sorting Intolerant From Tolerant (SIFT)	uncertain	0.004
MutationTaster	deleterious	1
Functional Analysis Through Hidden Markov Models (FATHMM)	uncertain	- 1.73
Deleterious Annotation of Genetic Variants Using Neural Networks (DANN)	deleterious	1
Logistic Regression-Based Ensemble Prediction Tool (MetaLR)	deleterious	0.82
PrimateAI (artificial Intelligence tool)	deleterious	0.8
BayesDel (deleteriousness meta-score)	deleterious	0.36
Splice AI tool	benign	0.01
Genomic Evolutionary Rate Profiling (GERP)	uncertain	6.16
Fitness Consequence Tool (FitCons)	deleterious	0.84

**Figure 4:**
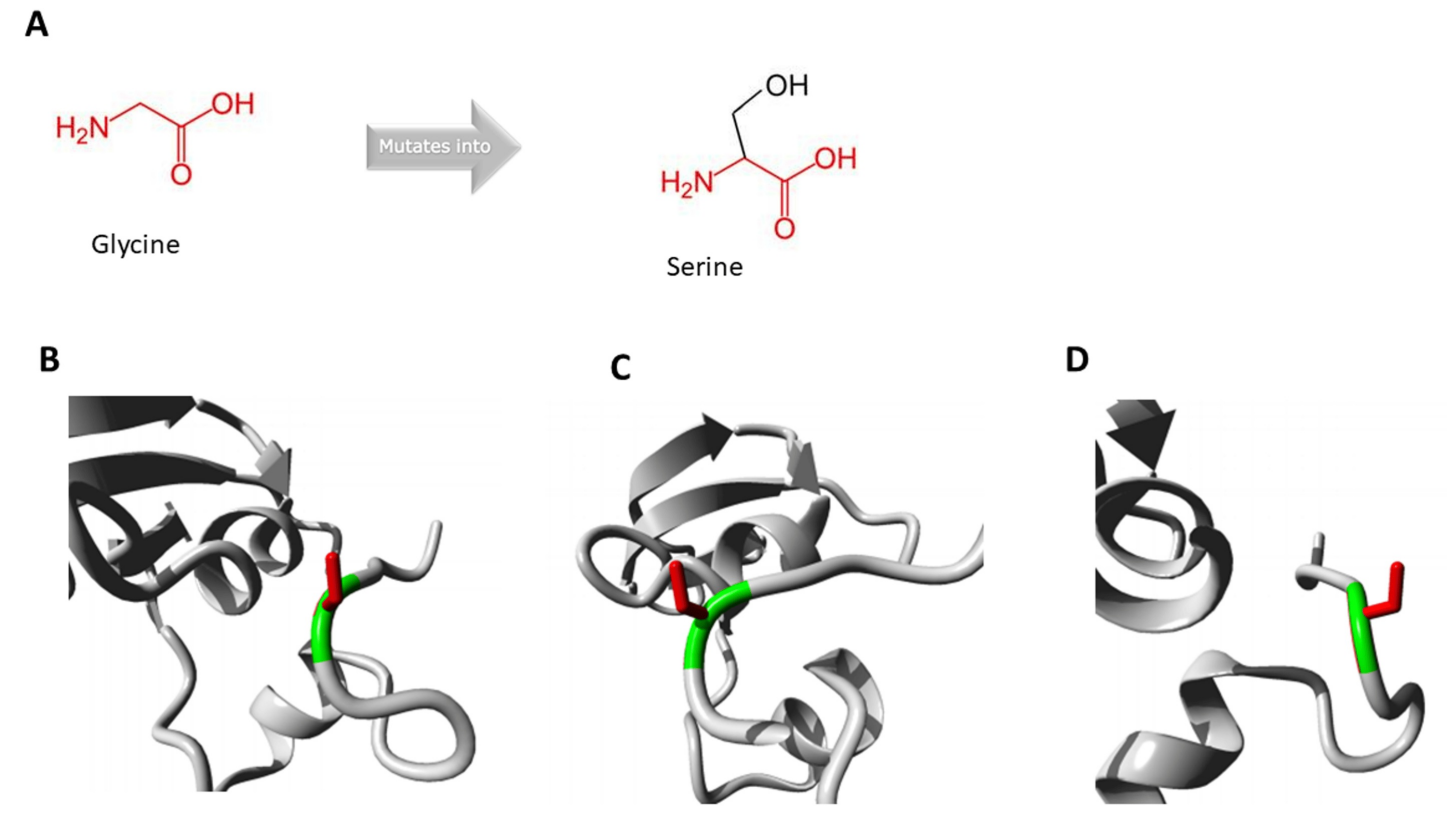
3D modeling of the FBN1 gene variant p.Gly1754Ser using HOPE online software Figure 4A shows the schematic structures of the original (left) amino acid glycine and the mutant (right) amino acid serine. The backbone, which is the same for each amino acid, is red. The side chain, unique for each amino acid, is black. Figures 4B-4D show a close-up of the mutation in the protein in ribbon presentation from different angles. The protein is gray. The side chains of both the wild-type and the mutant residue are green and red, respectively. HOPE: Have Our Protein Explained

Have Our Protein Explained (HOPE) in-silico software available online (https://www3.cmbi.umcn.nl/hope/) was used for 3D modeling of the variant. This software showed that the variant lies in a special domain, namely the transforming growth factor beta-binding domain, and that the torsion residues at this site suggest only glycine is flexible enough to make these torsion angles. Any other amino acid substitution will force the local backbone into an incorrect conformation and disturb the local structure (Figure 4, Video 1, Video 2).

**Video 1:** 3D modeling of the FBN1 gene variant p.Gly1754Ser using HOPE online software Close-up of the variant. The wild-type and mutant side chains are shown in green and red, respectively. The rest of the protein is shown in gray. HOPE: Have Our Protein Explained

**Video 2:** 3D modeling of the FBN1 gene variant p.Gly1754Ser using HOPE online software Close-up of the variant. The wild-type and mutant side chains are shown in green and red, respectively. The rest of the protein is shown in gray. The animation alternates between the wild-type side chain and the mutant side chain. HOPE: Have Our Protein Explained

The patient was therefore diagnosed to have WMS type 2, autosomal dominant variety, due to a pathogenic mutation in the FBN1 gene. The family underwent genetic counseling about the nature of the disease and the inheritance pattern. The risk to siblings was minimal because the parents were clinically unaffected. This was a sporadic case, but the risk of passing the disease to the progeny of the index child was 50%. Prenatal diagnosis or preimplantation genetic diagnosis can be useful in preventing recurrences in future generations. Atrial septal defect closure was planned. Life expectancy is normal.

## Discussion

To the best of the authors' knowledge, this is the first WMS type 2 patient with FBN1 gene mutation, published from India. The other clinical differential diagnoses in this case are isolated ectopia lentis, homocystinuria, MFS, sulfite oxidase deficiency, GLD, AMD, and Myhre syndrome [7]. Patients with isolated ectopia lentis (FBN1 or ADAMTSL4 gene mutation) do not have skeletal involvement [7]. MFS (FBN1 gene defect) patients have normal or tall stature, dolichostenomelia, pectus excavatum or carinatum, scoliosis, aortic root dilatation, mitral or tricuspid valve regurgitation, skin striae, and pes planus [7]. Homocystinuria (CBS gene defect) is characterized by ectopia lentis, myopia, tall stature, long limbs, scoliosis, pectus excavatum, thromboembolism, and developmental delay or mental retardation [7]. Sulfite oxidase deficiency (SUOX gene defect) is characterized by ectopia lentis and neurological involvement, such as ataxia, dystonia, neurological regression, and mental retardation [7]. Myhre syndrome (SMAD4 gene defect) is characterized by short stature, small hands, thick skin, mental retardation, microcephaly, midface hypoplasia, prognathism, blepharophimosis, square body shape, broad ribs, iliac hypoplasia, flattened vertebrae, thick calvaria, and congenital heart defects [7]. GLD, AMD, and WMS have overlapping clinical features that are collectively called acromelic dysplasias [2]. GLD, AMD, and WMS cause short height, small hands and feet, stiff joints, thick skin, delayed bone age, cone-shaped epiphyses, short bones, ovoid vertebral bodies, an internal notch of the second metacarpal, and an external notch of the fifth metacarpal [2]. GLD patients (FBN1, ADAMTSL2, or LTBP3 gene defect) have characteristic facial features, including a happy face with full cheeks, a short nose, hypertelorism, a long flat philtrum, a thin upper lip, tracheal stenosis that may require tracheostomy, hepatomegaly, and progressive cardiac valvular thickening, which leads to reduced life expectancy if left untreated. AMD patients have spherical faces, thick eyebrows, prominent eyelashes, large noses, a long philtrum, broad lips, small mouths, and thick voices [2]. WMS patients are similar to AMD patients, but lens dislocation is limited to WMS and absent in GLD and AMD patients [2].

The mutation-confirmed cases of WMS in the literature were reviewed. Faivre et al. described the clinical manifestations of WMS in 63 families. Their features included small spherical lenses (84%), dislocated lenses (73%), shortsightedness (94%), increased intraocular pressure (80%), cataracts (23%), short height (98%), small hands and feet (98%), and stiff joints (62%). Other manifestations included heart abnormalities (24%) and low intelligence (13%) [8]. In one of these cases, Faivre et al. identified a twenty-four base-pair deletion c.5074del24 in the FBN1 gene [9]. Kojuri et al. reported prolonged QTc interval in three of six patients, mitral valve prolapses in three of six patients, and one patient with valvular aortic stenosis [10]. Backer et al. described a family with an overlap between MFS and WMS with a twenty-base-pair deletion in exon 20 (c.2502-2513delTGAAAGTACTTT; p.Glu835-Leu838del). The proband was 172 cm tall, with an aortic root aneurysm suggesting MFS but a stocky build, round face, stiff joints, a small spherical lens, and a shallow anterior chamber suggestive of WMS. Other affected family members had typical features of MFS and not WMS. They also discussed other reported mutations in WMS, such as p.Gly214Ser (exon 6 reported by V. Cornier Daire, Universite´ Paris-Descartes, Paris), p.Arg1596Pro (exon 38 reported by G. M. Corson, Oregon Health and Science University, Portland, Oregon), and in-frame deletion of exons 9 to 11 [11]. Sengle et al. reported a family with WMS and a 7895 base-pair deletion with boundaries in introns 8 (IVS8-1207) and 11 (IVS11+1257). They introduced this mutation in a mouse model and demonstrated that the deletion abolished the binding site of fibrillin-1 to ADAMTS-like proteins, including ADAMTS10 [12]. Cecchi et al. described a 41-year-old African American with WMS and unusual complications of dilation of the aortic root and ascending aorta and sub-acute aortic dissection extending from the aortic root to the left subclavian artery. He had a lens extraction done at 7.5 years of age. He had prolonged QTc on the electrocardiogram, between 486 and 531 milliseconds. He showed heterozygosity for variant c.5242T>C or p.C1748R [13]. Wang et al. described a three-year-old patient with WMS who showed a novel variant p.C1748F in the FBN1 gene, the same site as in the patient reported by Cecchi et al. [14]. Newell et al. described a patient with WMS having short stature, dislocated lens, cervical artery dissection, and thoracic artery aneurysm caused by a mutation in exon 41 of the FBN1 gene (c.5161T>A; p.Cys1721Ser) [15]. Interestingly, different substitutions at this specific codon have been reported in an individual with geleophysic dysplasia and in an individual with skeletal, ocular, and cardiac features of MFS [16].

Sun et al. described two families with WMS and the variant p.Gly1754Ser, which was reported in our case [3]. In the first family, the index patient was a 2.3-year-old boy who was 83 cm in height (-2.5 SDS), had bilateral lens dislocation, had a birth weight of 2.6 kg, a birth length of 50 cm (normal), and had an affected mother with a height of 135 cm (-2.5 SDS) and bilateral lens dislocation. Both mother and child had small hands with stiffness in the interphalangeal joints. The mother had delayed bone age by four and two years at the chronological ages of seven and 14 years, respectively. Both mother and child were heterozygous for the p.Gly1754Ser variant seen in our patient. The second case reported by Sun et al. was of a 9.3-year-old boy of short stature (height 113 cm, <-3 SDS), lens subluxation, stiff hands, normal 2D echocardiography findings, a normal spine X-ray, and a hand X-ray showing delayed bone age. Yang et al. identified a Chinese family with WMS with three generations affected (a 68-year-old man, his 47-year-old daughter, and his 17-year-old grandson) with the novel intronic splice variant c.1327+1G>A. The index patient and his daughter were short in stature (163 cm and 150 cm, respectively) and had lens subluxations. However, the grandson was mildly affected (height 170 cm, mild lens opacity with no lens dislocation), demonstrating intrafamilial variability. Additionally, the presence of bilateral coloboma in the daughter was unusual [17]. Furthermore, in the Clinvar database, two more variants at this site are included as likely pathogenic for WMS or MFS, p.Gly1754Val (Clinvar database ID 2946404) and p.Gly1754Asp (Clinvar database ID 2570580). Clinical details of these cases are not provided in the database.

## Conclusions

WMS is a genetic disorder with the autosomal dominant form caused by a mutation in the FBN1 gene. The disorder is characterized by short height, delayed bone age, small hands and feet, stiff joints, lens dislocation, normal intelligence, heart defects such as aortic root dilatation, and prolonged QTc interval. Clinical and molecular confirmation of WMS is essential for genetic counseling, management, and prevention of recurrences in future generations by prenatal diagnosis or preimplantation genetic diagnosis. Exome sequencing provides a definitive diagnosis by identification of the FBN1 gene mutation. In-silico analysis and bioinformatic analysis elucidate the molecular mechanism of the mutation. Clinical, radiological, molecular, and in-silico studies for an illustrative case and the published cases in medical literature were discussed.
